# Dynamic Responses of a Metro Train-Bridge System under Train-Braking: Field Measurements and Data Analysis

**DOI:** 10.3390/s20030735

**Published:** 2020-01-29

**Authors:** Xuhui He, Kehui Yu, Chenzhi Cai, Yunfeng Zou, Xiaojie Zhu

**Affiliations:** 1 School of Civil Engineering, Central South University, Changsha 410075, China; xuhuihe@csu.edu.cn (X.H.); 164801022@csu.edu.cn (K.Y.); yunfengzou@csu.edu.cn (Y.Z.); 2 National Engineering Laboratory for High Speed Railway Construction, Changsha 410075, China; 3 Joint International Research Laboratory of Key Technology for Rail Traffic Safety, Changsha 410075, China; 4 Guangzhou Metro Corporation, Ltd., Guangzhou 510000, China; zhuxiaojie@gzmtr.com

**Keywords:** elevated metro line, train-bridge system, acceleration, Choi-Williams distribution, wavelet coherence

## Abstract

This paper focuses on the dynamic responses of a metro train–bridge system under train-braking. Experiments were performed on the elevated Metro Line 21 of Guangzhou (China). A continuous, three-span, rigid-frame bridge (42 m + 65 m + 42 m) and a standard B-type metro train were selected. The acceleration signals were measured at the center-points of the main span and one side-span, and the acceleration signals of the car body and the bogie frame were measured simultaneously. The train–bridge system’s vibration characteristics and any correlations with time and frequency were investigated. The Choi–Williams distribution method and wavelet coherence were introduced to analyze the obtained acceleration signals of the metro train–bridge system. The results showed that the Choi–Williams distribution provided a more explicit understanding of the time–frequency domain. The correlations between different parts of the bridge and the train–bridge system under braking conditions were revealed. The present study provides a series of measured dynamic responses of the metro train–bridge system under train-braking, which could be used as a reference in further investigations.

## 1. Introduction

As a result of rapid urbanization, the continuously increasing population and expanding areas of most urban cities are increasing the demand for transportation in cities. The urban metro system is an effective way of satisfying the need for public transport. Thus, vigorous development of metro systems occurred globally, especially in China. According to recent statistics, the total length of operating metro lines was about 6000 km throughout 41 cities in China at the end of 2019. Aside from the general underground metro, the elevated metro was widely adopted in recent years in China due to its short construction period and low-cost advantages, especially in cities such as Beijing, Shanghai, and Guangzhou. Currently, more and more cities are increasing the maximum operational speeds of metro lines to adapt to the needs of city development.

The dynamic impacts of moving trains on viaducts influence the working state and service reliability of bridges. Furthermore, the responses of bridges can affect the stability and safety of moving vehicles. Thus, it is necessary to study train–bridge dynamic interactions to ensure safety and smooth operation. An abundance of studies regarding theoretical analyses and numerical simulations were conducted throughout the last few decades, with both theoretical and numerical models of the train–bridge system being expanded from an one-dimensional approach to a multidimensional approach due to the development of mathematics, mechanics, and computer technology [1,2,3]. However, due to the complex interactions of the train–bridge system, various simplifications were always adopted during the theoretical analyses and numerical simulations. Thus, field measurement remains an indispensable method to investigate the dynamic characteristics of the train–bridge system. Xia et al. [4] measured the dynamic responses of a high-speed train–bridge system in order to validate theoretical models containing simplifications of an actual situation. Zhai et al. [5] investigated the long-term service performance of a China Railway high-speed train traveling at 350 km/h over two months on-track. Sayed et al. [6] investigated and assessed the movement behavior of a simple supported high-speed train railway bridge using measured acceleration data. 

Generally, the complexity of train–bridge systems and multiple vibration excitation sources produce test signals with nonlinear and nonstationary characteristics. Moreover, several noise components generally accompany these test signals. Thus, an appropriate method must be chosen to obtain vibration characteristics of the dynamic response of a train–bridge system when trains are running. The wavelet theory was proven to be effective in solving nonstationary problems, with denoising advantages [7,8,9]. However, many wavelet bases are present in the wavelet transform, each with their own tradeoff between the time and frequency parameters. Therefore, the Choi–Williams distribution (CWD) was proposed to improve the representation of a time-varying, nonstationary signal for both the time and frequency domains. Currently, the CWD is increasingly applied to nonstationary signal analyses in the fields of aerospace, communications, biomedicine, and sonar, as it suppresses cross-term interference [10,11,12]. However, available applications of CWD with regard to the nonstationary signals of a train–bridge system are limited. 

There are some investigations related to the effects of braking load on the stability and safety of a moving vehicle [13,14]. The complex train-bridge dynamic interactions under the train’s braking also have the potential to affect the train’s running performance. Experimental studies regarding the dynamic responses of a metro train–bridge system are still lacking, with even fewer field measurements concerning train-braking on bridges. In this work, we conducted a series of field measurements to investigate the dynamic responses of a metro train–bridge system under train-braking. A continuous, three-span, rigid-frame bridge (42 m + 65 m + 42 m) and a standard B-type metro train were selected. Experiments were performed using the elevated Metro Line 21 of Guangzhou (China). The CWD method was introduced to analyze the obtained acceleration signals of the metro train–bridge system to gain more explicit comprehension of the time–frequency domain. Wavelet coherence was adopted to reveal the correlation between different parts of the bridge and the train–bridge system, thereby providing a series of measured dynamic responses of the train–bridge system under train-braking, which could be used as a reference for further investigations regarding metro train–bridge systems.

## 2. Field Measurement

**Figure 1:**
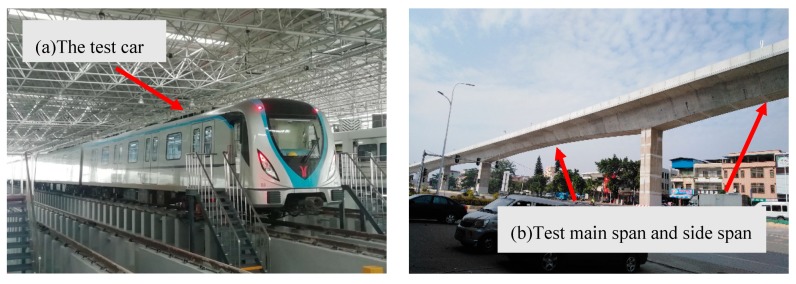
Photo of the selected train and bridge: (**a**) the B-type metro train; (**b**) the continuous, three-span, rigid-frame bridge (42 m + 65 m + 42 m).

**Figure 2:**
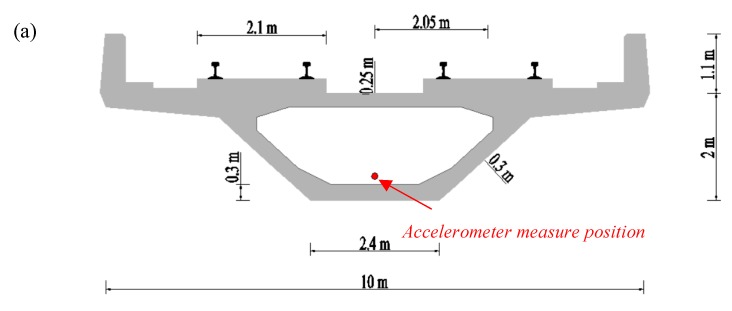

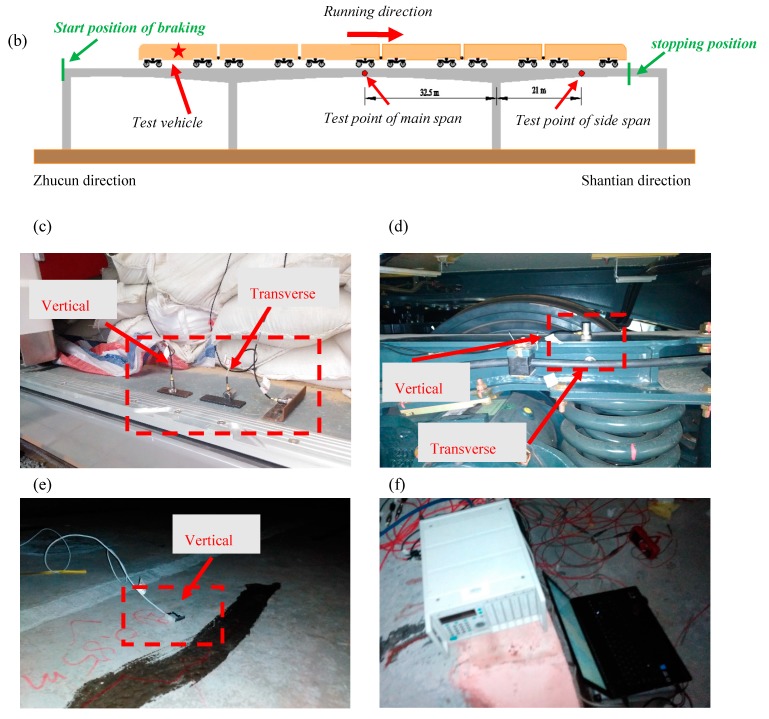
Field test set-up: (**a**) schematic diagram of the box girder; (**b**) schematic diagram of the experiment; (**c**) measurement points on the train body; (**d**) measurement points on the bogie frames; (**e**) measurement points on the bridge; (**f**) the HBM data collection system.

The bridge under investigation is a continuous, concrete, rigid-frame, box-girder bridge with three spans, located between Zhucun Station and Shantian Station of Guangzhou Metro Line 21, as illustrated in Figure 1. The length of the main span is 65 m and the two side spans are each 42 m long. The height of the box girder varies according to a quadratic parabola model, but the cross-sectional dimensions of the center-points of both the main span and two side spans are the same, as shown in Figure 2. The widths of the bridge deck and the bottom slab are 10 m and 2.4 m, respectively. Two track systems are arranged symmetrically according to the central line of the cross-section. The distance between the central lines of the two track systems is 2.05 m.

A standard B-type metro train was used in this experiment, as demonstrated in Figure 1, which consists of six cars, including two trailer cars and four motor cars. The head and tail are trailer cars, and four motor cars are arranged between the two trailer cars. The maximum speed of a B-type metro train is 120 km/h. During the experiments, each carriage was loaded with 20 tons of sandbags in order to simulate the sum of seated passenger loads at maximum seating capacity and the standing passenger loads at the density of six passengers per square meter. The length of a B-type metro vehicle is 19 m and the length of the whole train is about 120 m.

Two accelerometers (type: DH1A111E, 1 mV/g sensitivity, 50 g full-scale) were mounted onto the body of the car (back-left) and bogie frame (back-left) of the last vehicle, in order to obtain the vertical and transverse acceleration signals of the train. The dynamic response data were collected using a 24-bit intelligent acquisition and signal-processing system (type: DH5922N). The sampling frequency was 2000 Hz, and the vertical and transverse acceleration signals of the center-points of the main span and one side-span were measured using SDI Model 2210 accelerometers (4 mV/g sensitivity, ±2 g full-scale). The accelerometers were also installed at the inner bottom of the box girder, as illustrated in Figure 2. The HBM (Hottinger Baldwin Messtechnik GmbH) data collection system (MGCPlus) with a sampling frequency of 200 Hz was used to measure the dynamic responses of the test bridge.

The speed of the test train was controlled throughout the experiments on the continuous rigid-frame bridge. Both cases of constant speed and braking were conducted. In the case of constant speed, the train passed through the bridge at a constant speed of 120 km/h. In the braking simulation, the train’s direction was from Zhucun toward Shantian; the test train had an initial speed of 70 km/h when braking was initiated, i.e., when it arrived at the starting mileage position on the continuous, three-span, rigid-frame bridge. The maximum deceleration of the metro train should be smaller than 1.2 m/s^2^, according to Chinese standards [15]. The braking process took approximately 12.5 s, and the braking distance was approximately 139 m. The length of the whole train (about 120 m) was less than the length of the bridge (149 m), meaning that the whole train could stop on the bridge, as illustrated in Figure 2b.

## 3. Signal Processing Method

The train–bridge interaction system includes two complex components, i.e., the vehicle and the bridge, resulting in the obtained signals possessing nonlinear and nonstationary properties. Moreover, the measured signals are often mixed with a great deal of noise due to the influence of the complex structure of the vehicle and multiple vibration excitation sources. The Choi–Williams distribution method and wavelet coherence were used to analyze the acquired acceleration signals from the metro train–bridge system. A classical frequency analysis method (fast Fourier transform) and a time–frequency analysis method (wavelet theory) are briefly discussed in this section.

### 3.1. The Fast Fourier Transform

Fast Fourier transform (FFT) is one of the most important numerical algorithms; it is widely used in signal analysis and is involved in transforming original signals from the time domain to the frequency domain, thus allowing the spectral characteristics of signals to be extracted. FFT can be expressed as follows [16]:

(1) $$X(k)=\sum_{n=0}^{N-1}x(n)W_{N}^{kn},$$

where $W_{N}^{}=e^{-j(2n/N)}$ and *N*=length [x(n)]. However, the time-varying spectral characteristics of the measured signals cannot be obtained by applying FFT to the total time domain.

### 3.2. Wavelet Theory

Wavelet transform expands on the basis of Fourier analysis to identify the time–frequency characteristics of signal processing. Wavelet transform is widely applied as an excellent time–frequency analysis tool to analyze nonstationary signals in many areas of research [17,18]. There are two types of wavelet transform, namely, continuous wavelet transform (CWT) and discrete wavelet transform (DWT). The continuous wavelet transform of function *f*(*t*) can be defined as follows [19]:

(2) $$w_{f}\left(a,b\right)=\frac{1}{\sqrt{a}}\int_{-\infty }^{\infty }f\left(t\right)\psi \left(\frac{t-b}{a}\right)dt,a\in R^{+}\&b\in R,$$

where ψ(t) is the mother wavelet with a scaling factor *a* and the shift factor *b*.

In order to eliminate redundant components in the CWT due to the continuous variation of the scale and shift factors, high-pass and low-pass filters were adopted into the DWT to decompose the original signal into two parts, namely, a low-frequency part (the approximated component) and a high-frequency part (the detailed component). Thus, the decomposition of a signal using DWT can be described as follows:

(3) $$S_{j}(t)=a_{j+1}(t)+d_{j+1}(t),$$

where the subscript j represents the amount of decomposition, $S_{j}(t)$ represents the original signals, and $a_{j+1}(t)$ and $d_{j+1}(t)$ are the approximated and detailed components, respectively.

Many environmental impacts during data acquisition are unavoidable; therefore, measured signals are often mixed with a great deal of noise, which may lead to unreasonable results. Thus, wavelet denoising is widely used in signal processing with proven efficacy. The preprocessed procedure using wavelet denoising reduced the effects of the noise components in the original signal. The wavelet denoising process involved three steps [20]: (1) choosing the wavelet basis and determining the amount of decomposition, (2) using a soft threshold for each decomposition level to remove noise components, and (3) reconstructing the signal using the processed wavelet coefficients. The algorithms of the soft threshold ($T_{soft}$) can be expressed as follows:

(4) $$T_{soft}=sgn\left(x\right)\cdot \left(\left|x\right|\cdot Thr\right),$$

where x represents the wavelet coefficient, and $Thr=\sigma \sqrt{2\log\left(N\right)}$ is the universal “VisuShrink” threshold (where σ is the noise standard variance, and N is the size or length of the signal). The noise signal usually exists in the detailed components; thus, the processed details and non-processed approximations are used for signal reconstruction. The details are obtained in the decomposition processing of the original signal and sorted into different frequency bands.

### 3.3. Choi-Williams Distribution Method

Although wavelet theory is widely adopted in time–frequency analyses, a proper wavelet basis should be chosen carefully in order to obtain explicit time–frequency results. There are many wavelet bases, all with their own advantages; some wavelet bases obtain good results in the frequency domain, whereas other wavelet bases are advantageous in regard to the time domain. Due to the existence of different dominating frequencies in the train and bridge acceleration signals, it was not easy to find a uniform wavelet basis for the train–bridge interaction system. Thus, the Choi–Williams distribution (CWD) was used to minimize cross-term effects and to maintain a high-quality time–frequency resolution. The CWD algorithm for a nonstationary signal is expressed as follows [21]:

(5) $$CWD_{x}\left(t,f\right)=\iint \sqrt{\frac{\sigma }{4\pi \tau^{2}}}\exp\left(-\frac{\sigma t^{2}}{4\tau^{2}}\right)x\left(\mu +\frac{\tau }{2}\right)x^{*}\left(\mu -\frac{\tau }{2}\right)e^{-j2\pi f\tau }d\mu d\tau ,$$

where τ represents the time-shift parameter, σ represents the scale factor, μ represents the partial time, and the superscript * denotes the complex conjugate. It should be noted that the exponential kernel function $g(\theta ,\tau )=\exp(-\theta^{2}\tau^{2}/\sigma )$ (where θ represents the frequency offset parameter) is adopted in Eqution (5). The exponential kernel function g(θ,τ) satisfies the conditions of g(0,τ)=g(θ,0)=1, g(0,0)=1, and g(θ,τ)<1(θ≠0,τ≠0). The inhibitory effect of cross-term becomes stronger when σ decreases; therefore, the exponential kernel function could contribute to a high-quality time–frequency performance by suppressing cross-term interference.

### 3.4. Wavelet Coherence

Wavelet coherence was adopted to investigate the train–bridge interaction behaviors during braking. Although the sampled frequencies of the train and bridge acceleration signals were different, the dominating frequencies of both these acceleration signals were generally less than 50 Hz. Thus, the measured acceleration signals of the train components (car body and bogie frame) were resampled from 2000 Hz to 200 Hz. Then, the wavelet coherence was applied to process the train and bridge acceleration signals respectively during braking. One particular wavelet, the Morlet wavelet, was adopted here, which is defined as follows [22,23]:

(6) $$\psi_{0}\left(\eta \right)=\pi^{-1/4}e^{i\omega_{0}\eta }e^{-\frac{1}{2}\eta^{2}},$$

where $\omega_{0}$ and η are dimensionless frequency and dimensionless time, respectively.

By applying the convolution to a time series $x_{n}(n=1,\ldots ,N)$ with scaled and normalized wavelets, the local phase can be expressed as follows:

(7) $$W_{n}^{X}\left(s\right)=\sqrt{\frac{\delta t}{s}}\sum_{n’=1}^{N}x_{n’}\psi_{0}\left[\left(n’-n\right)\frac{\delta t}{s}\right].$$

The wavelet coherence of two time series, $x_{n}$ and $y_{n}$, was adopted to reveal the relationships between them. This is defined as follows:

(8) $$R_{n}^{2}\left(s\right)=\frac{{\left|S\left(s^{-1}W_{n}^{XY}\left(s\right)\right)\right|}^{2}}{S\left(s^{-1}{\left|W_{n}^{X}\left(s\right)\right|}^{2}\right)\cdot S\left(s^{-1}{\left|W_{n}^{Y}\left(s\right)\right|}^{2}\right)},$$

where $W^{XY}=W^{X}W^{Y*}$ (the superscript * denotes complex conjugation) represents the cross wavelet transform of two time series, and S (which can be expressed as $S(W)=S_{scale}(S_{time}W_{n}(s))$) represents the smoothing operator used to balance resolution and significance. For the Morlet wavelet, a suitable smoothing operator is given as follows:

(9) $${S_{time}\left(W\right)|}_{s}={\left(W_{n}\left(s\right)*c_{1}^{\frac{-t^{2}}{2s^{2}}}\right)|}_{s},{S_{scale}\left(W\right)|}_{s}={\left(W_{n}\left(s\right)*c_{2}\Pi \left(0.6s\right)\right)|}_{n},$$

where $c_{1}$ and $c_{2}$ are normalization constants, and Π represents the rectangle function.

**Figure 3:**
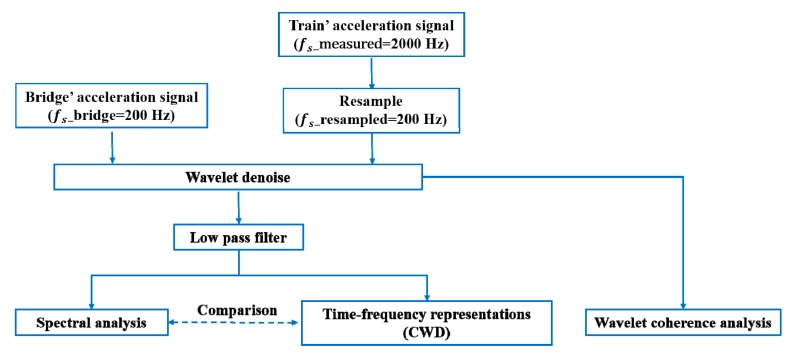
Schematic diagram of the time-frequency test signal analysis process from the train-bridge system.

The analysis process of the original acceleration signals of the train–bridge system is schematically illustrated in Figure 3. The acquired acceleration signals of the train at 2000 Hz were resampled to 200 Hz; then, FFT was applied to the acceleration signals of the train–bridge system for spectral analysis. Through implementation of the soft thresholding process, the denoised versions of the original signals were used for wavelet coherence analysis to correct the acceleration signals of the train–bridge system in the time–frequency domain. A low-pass filter was used for the denoised versions of the original signals to obtain time–frequency representations of the train and bridge acceleration signals using CWD. The cut-off frequencies of the low-pass filter for the acceleration signals of the bridge, car body, and bogie frame were 30, 30, and 10 Hz, respectively. The correlations of the dynamic response of the train–bridge system were revealed by applying wavelet coherence analysis. 

### 3.5. Comparison

**Figure 4:**
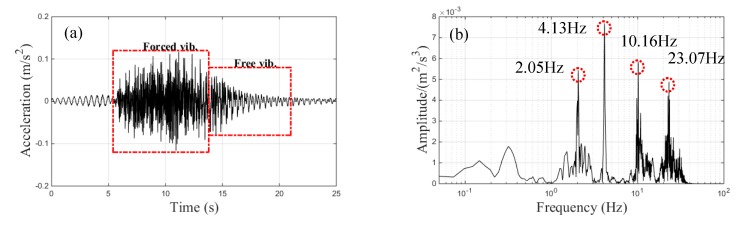
Vertical acceleration signals of the center-point of the main span under a train speed of 120 km/h: (**a**) resampled data; (**b**) fast Fourier transform (FFT) analysis results.

**Figure 5:**
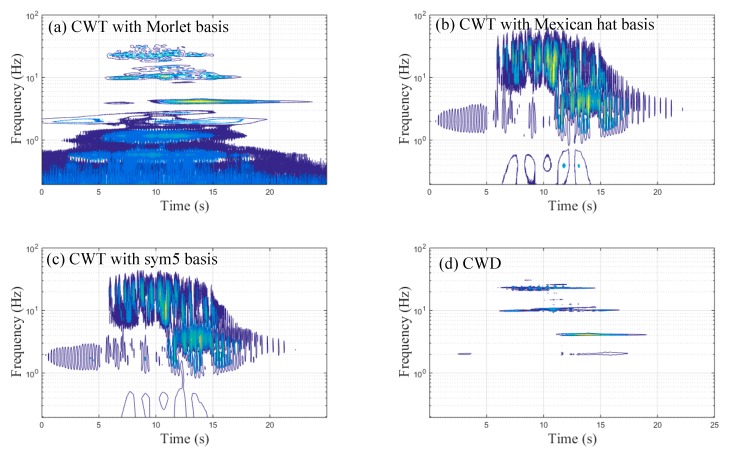
The time–frequency representation results of the center-point’s acceleration signals of the main span under a train speed of 120 km/h with respect to different methods: (**a**) continuous wavelet transform (CWT) with the Morlet basis; (**b**) CWT with the Mexican-hat basis; (**c**) CWT with the sym5 basis; (**d**) Choi–Williams distribution (CWD).

The vertical acceleration signals from the center-point of the main span under the train speed of 120 km/h are shown in Figure 4a. The forced vibration part (from the train movement on the bridge) was during the 5.8–13.8 s period, and the free vibration part (after the whole train passed along the bridge) was during the 13.8–25 s period. The corresponding spectral results using FFT are shown in Figure 4b, which shows four obvious peaks at 2.05, 4.13, 10.16, and 23.07 Hz in the frequency domain. However, satisfactory time–frequency characteristics could not be obtained due to the time-varying acceleration signals. Many wavelet bases exist in wavelet theory, each with their own advantages in the time–frequency representation. CWT results were compared by adopting the Morlet basis, the Mexican-hat basis, and the sym5 basis, and the Choi–Williams distribution results were obtained, as illustrated in Figure 5. The results shown in Figure 5 are from the same time-domain signal of the center-point’s acceleration of the main span under a train speed of 120 km/h. Four clear frequency peaks around 2, 4, 10, and 23 Hz were observed, as shown in Figure 5d, which were nearly identical to the FFT analysis results (as shown in Figure 4b). Moreover, these frequency peaks were identified in the time domain simultaneously. However, these frequency peaks were not clearly in Figure 5b,c. In Figure 5a, there were three obvious frequency bands (about 4 Hz, 10 Hz and 23 Hz). However, the extensive low-frequency content in Figure 5a lasted about the whole measured acceleration signals; such a phenomenon was because of the low resolution of the CWT with Morlet basis in the range of 0-1 Hz rather than the vibration of the bridge. Therefore, the time–frequency representation results (TFRs) of the CWD showed more explicit resolution in the time–frequency domain, and CWD was adopted to obtain the TFRs, as discussed in the next section.

## 4. Results and Discussion

### 4.1. Dynamic Characteristics of the Continuous Rigid Frame Bridge

**Figure 6:**
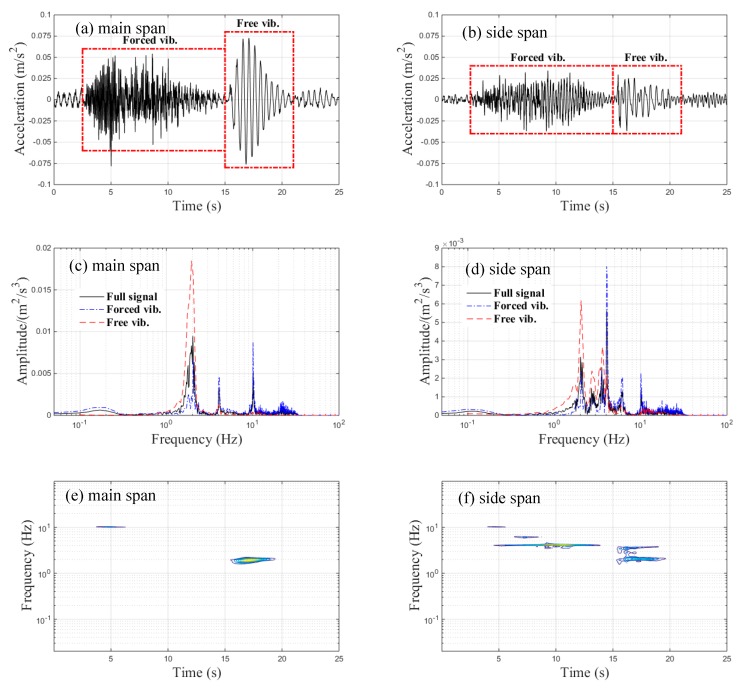
Vertical acceleration signals of the bridge during braking: (**a**) time-history data of the main span; (**b**) time-history data of the side span; (**c**) FFT results of the main span; (**d**) FFT results of the side span; (**e**) time–frequency representation results (TFRs) of the main span; (**f**) TFRs of the side span.

The vertical acceleration trends of the continuous, three-span, rigid-frame bridge measured at the center-points of the main span and side span during braking are shown in Figure 6a,b, respectively. The free vibration part of the braking case referred to the whole train stopping on the bridge after braking; these corresponding spectral results obtained via FFT are demonstrated in Figure 6c,d, respectively. The maximum amplitudes of the main and side spans’ measured accelerations were 0.07 m/s^2^ and 0.04 m/s^2^, respectively. The maximum amplitude of the accelerations at the center-point of the main span occurred in the free vibration part, as illustrated in Figure 6a. As seen in Figure 6c, only one peak frequency at about 1.95 Hz was observed in the free vibration part of the main span’s acceleration signal, while three frequency peaks at about 2.05, 4.1, and 10.11 Hz were observed in the forced vibration part of the main span’s acceleration signal, as illustrated in Figure 6c. The FFT results of the side span’s acceleration signal showed two frequency peaks (at about 1.95 Hz and 3.6 Hz) and four frequency peaks (at about 2.051, 4.053, 6.055, and 10.11 Hz) corresponding to the free and forced vibration parts, respectively, as shown in Figure 6d. The dominant frequency of the free vibration part is the natural frequency for bridges [24]. Therefore, the first and second fundamental vertical frequencies of the continuous, three-span, rigid-frame bridge corresponding to approximately 2 and 4 Hz, respectively, were obtained by performing spectrum analysis of the measured acceleration data. The first fundamental frequency dominated the free vibration part of the main span, whereas both the first and the second fundamental frequencies dominated the free vibration part of the side span. Notably, the predominant frequencies and their corresponding FFT full-signal amplitudes of both the main and side spans were different from the FFT results seen from the forced and free vibration parts. Figure 6e,f show the main and side span TFRs, respectively. Figure 6e shows that the dominant frequency was concentrated around 2 Hz during the free vibration, but no obvious dominant frequency was observed during the forced vibration of the main span. As shown in Figure 6f, the dominant frequency ingredients corresponding to the forced and free vibration parts were about 2 and 4 Hz, respectively.

**Figure 7:**
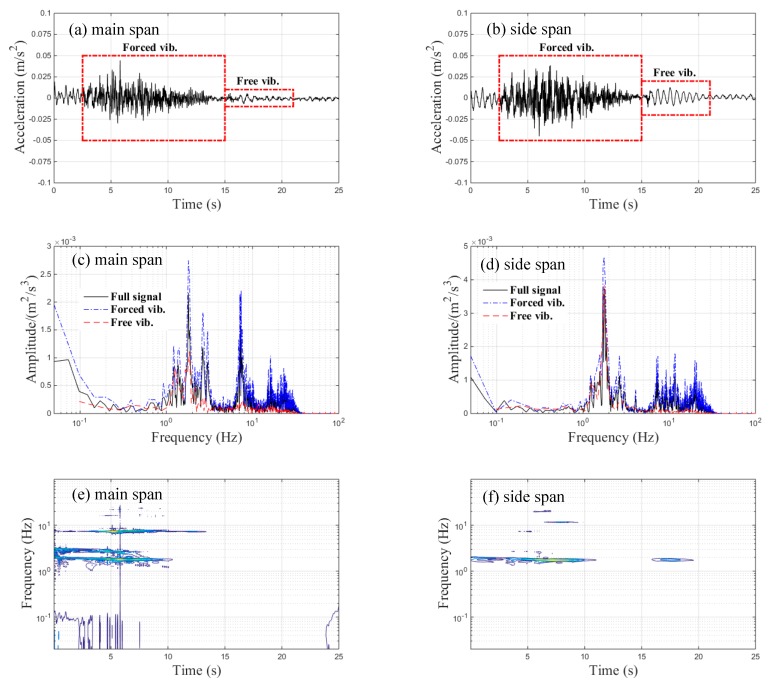
Transverse acceleration signals of the bridge during braking: (**a**) time-history data of the main span; (**b**) time-history data of the side span; (**c**) FFT results of the main span; (**d**) FFT results of the side span; (**e**) TFRs of the main span; (**f**) TFRs of the side span.

The time-history data regarding the transverse accelerations of the bridge measured at the main and side spans’ center-points under braking are shown in Figure 7a,b, respectively. Both the main and side span maximum amplitudes of transverse accelerations approximated to 0.04 m/s^2^. The measured maximum transverse accelerations of the different spans were far less than the threshold value of 1.4 m/s^2^, which is required under Chinese standards [25]. Moreover, the amplitudes of the transverse accelerations in both of the free vibration parts were very small. This is because that train’s braking force mainly acts on the vertical and longitudinal direction of the bridge, while the transverse vibration of the bridge is mainly affected by the moving train load. The corresponding spectral results obtained via FFT are demonstrated in Figure 7c,d, respectively. The FFT results of the main span’s acceleration signal show that there were three frequency peaks (about 1.81, 2.69, and 7.13 Hz) and only one frequency peak (about 1.86 Hz) corresponding to the forced and free vibration parts, respectively, whereas the dominant frequencies of the side span existed at 1.75 Hz and 7–15 Hz for the forced vibration and 1.75 Hz for the free vibration. The spectral analysis results of the full transverse acceleration signal of both spans were similar to their corresponding forced vibration spectral results. The TFRs of the transverse acceleration signals with respect to the main span and side span are illustrated in Figure 7e,f, respectively. Three frequencies existed in the forced vibration part of the main span’s transverse acceleration signals, as shown in Figure 7e, but only one frequency was observed with respect to the forced vibration part of the side span’s transverse acceleration signals, as seen in Figure 7f. These results indicated that CWD provided a better resolution of the time–frequency domain.

### 4.2. Dynamic Characteristics of the Metro Train

**Figure 8:**
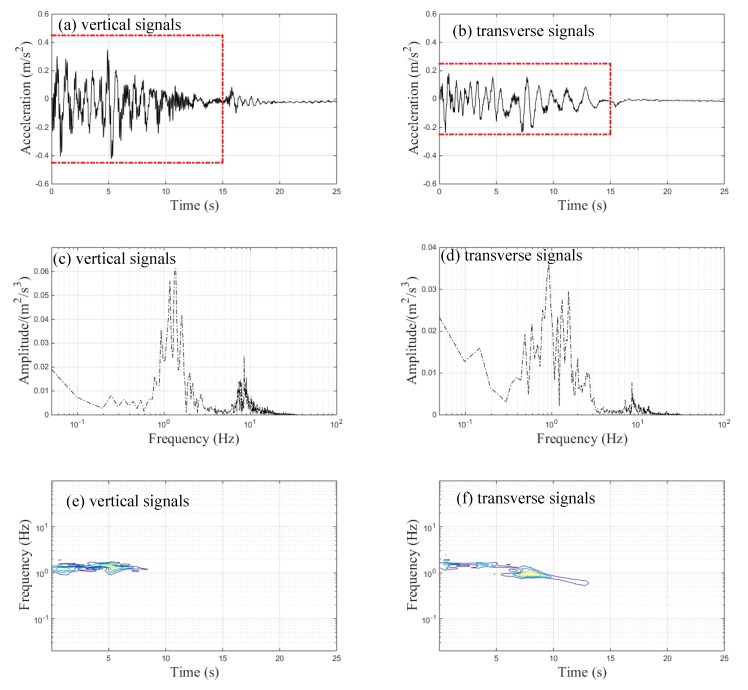
Acceleration signals of the car body during braking: (**a**) time-history data of vertical signals; (**b**) time-history data of transverse signals; (**c**) FFT results of vertical signals; (**d**) FFT results of transverse signals; (**e**) TFRs of vertical signals; (**f**) TFRs of transverse signals.

Figure 8a,b illustrate the vertical and transverse acceleration signals of the car body in the braking case. The acceleration signals were approximately zero during the 15–25 s period, indicating that the train completely stopped on the bridge. Thus, spectral analysis was only applied to the acceleration signals during the 0–15 s period. The maximum vertical and transverse acceleration signals of the car body were 0.42 m/s^2^ and 0.24 m/s^2^, respectively. The maximum vertical acceleration value of the car body was about 1.8 times larger than the maximum transverse acceleration value of the car body. Nevertheless, the maximum measured acceleration signals of the car body were far lower than the threshold value of 2.5 m/s^2^, as required by Chinese standards [26], indicating that the B-type metro train possesses excellent stability on elevated metro lines.

Figure 8c,d show two distinct frequencies around 1 and 8.5 Hz in both the vertical and the transverse acceleration signals of the car body. The first dominant frequency (around 1 Hz) was caused by the natural vibration frequency of the suspension system. The second dominant frequency (around 8.5 Hz) was close to the first-order natural vibration frequency of vertical bending (vertical acceleration) and torsional vibration (transverse acceleration) of the car body [5]. Figure 8e,f show that the vibration energy of the acceleration signals of the car body was mainly concentrated on the first predominant frequency, indicating that the vibration of the car body generated by the suspension system provided the dominant effect during braking.

**Figure 9:**
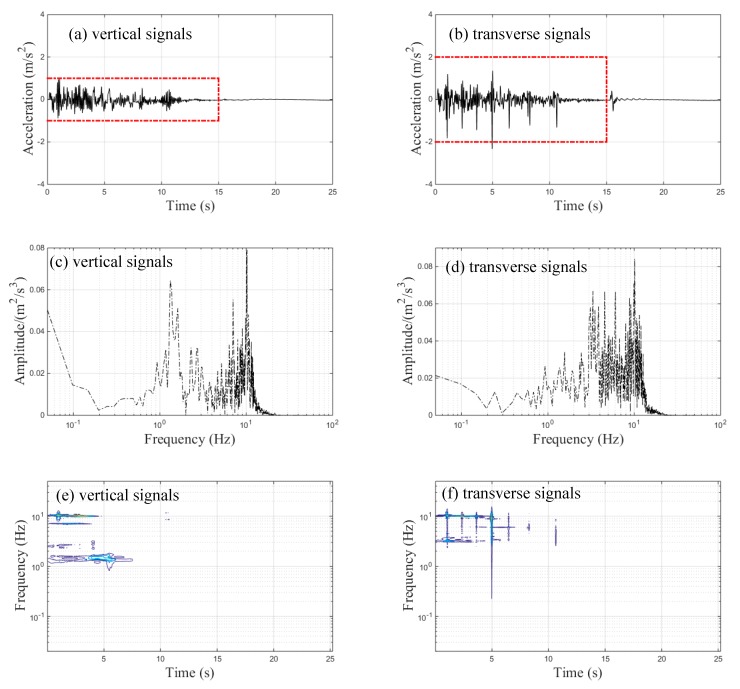
Acceleration signals of the bogie frame during braking: (**a**) time-history data of vertical signals; (**b**) time-history data of transverse signals; (**c**) FFT results of vertical signals; (**d**) FFT results of transverse signals; (**e**) TFRs of vertical signals; (**f**) TFRs of transverse signals.

Figure 9a,b illustrate the vertical and transverse acceleration signals of the bogie frame during braking. The maximum amplitudes of the vertical and transverse acceleration signals of the bogie frame were 0.96 m/s^2^ and 2.33 m/s^2^, respectively, which were far lower than threshold value of 8 m/s^2^, as required by Chinese standards [27,28], thereby indicating that the B-type metro train was stable when braking at the train’s initial speed of 70 km/h. As seen in Figure 9c, there were two distinct frequencies at 1.32 and 10 Hz regarding the vertical acceleration of the bogie frame, but there were several peaks in the range of 3–10 Hz regarding transverse acceleration. The bogie frame’s vertical and transverse signal TFRs are illustrated in Figure 9e,f, respectively. The dominant frequencies of the bogie frame’s vertical and transverse signals were similar to the FFT results. Moreover, several instantaneous peaks in the time-history data of the bogie frame’s transverse acceleration signals (Figure 9b) were obviously displayed in the TFRs (Figure 9f). These results indicated that CWD provided better resolution in the time–frequency domain.

### 4.3. Correlation of Different Acceleration Signals

Although localization of instantaneous dynamic characteristics of the train–bridge system was identified through the time–frequency representation results using CWD, the relationship between the train and bridge dynamic responses could not be revealed. Moreover, the relationship between the train’s dynamic responses and the bridge’s dynamic responses is rarely investigated. Thus, wavelet coherence was adopted to reveal the nature of the interactions between the train and bridge regarding the time–frequency domain based on the measured data. The relationship between the main span and the side span was also investigated. The descriptions for the four sets of signals regarding the train–bridge system are exhibited in Table 1. In order to clearly distinguish between significant and non-significant correlations of the signals in each set, a carefully selected color scheme was used. A significant correlation is presented in yellow, while a non-significant correlation is presented in blue. The wavelet coherence index was between 0 and 1, with a larger index number representing a more significant correlation. 

**Figure 10:**
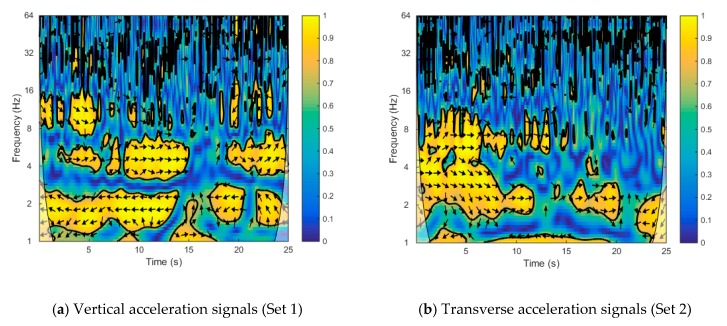
Wavelet coherence between the main span’s signals and the side span’s signals: (**a**) vertical acceleration signals (Set 1); (**b**) transverse acceleration signals (Set 2). The arrows represent the relative phase relationship, where a right-pointing arrow represents in-phase and a left-pointing arrow represents anti-phase.

**Figure 11:**
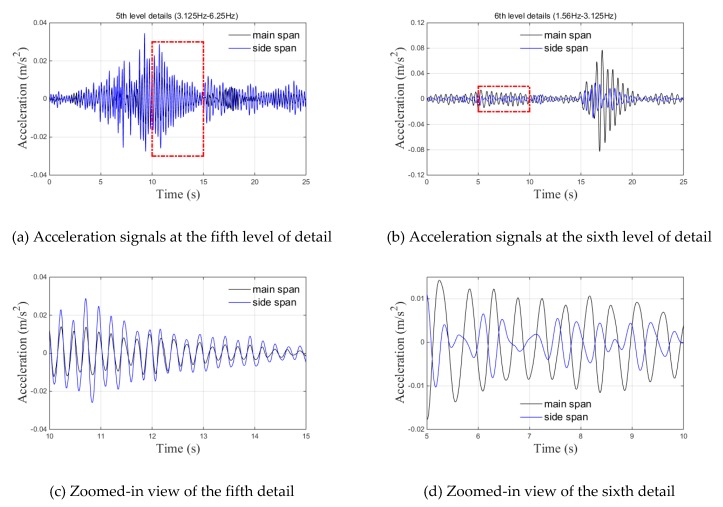
Details of the fifth and sixth decomposition levels regarding the vertical acceleration signals in Set 1: (**a**) vertical acceleration signal details at the fifth level; (**b**) vertical acceleration signal details at the fifth level; (**c**) zoomed-in view of the fifth detail; (**d**) zoomed-in view of the sixth detail.

The wavelet coherence of the main and side span acceleration signals is illustrated in Figure 10. As shown in Figure 10a, the significant regions of Set 1 mainly existed around 2 and 4 Hz, which corresponded to the first and second vertical fundamental frequencies of the bridge, respectively. The arrows represent the relative phase relationship, where a right-pointing arrow represents in-phase and a left-pointing arrow represents anti-phase [22,23]. The arrows show an in-phase correlation at a frequency of around 4 Hz and an anti-phase correlation at frequency of around 2 Hz, respectively. The observation that the phase angles were constant across these scales in the significant areas indicated that there was a constant time lag due to the physical mechanism of signal propagation from the main span to the side span. According to the discrete wavelet transform theory, the measured acceleration signals in Set 1 were decomposed into six frequency bands. The details of these two vertical acceleration signals from the bridge, which corresponded with the frequency bands [1.56, 3.125 Hz] (sixth level) and [3.125, 6.25 Hz] (fifth level), were chosen for an in-depth analysis, as shown in Figure 11a,b. The zoomed-in views of these details are shown in Figure 11c,d. Almost identical periods and phases of these two details led to an in-phase correlation, as shown in Figure 11c, but opposite phases and different periods of these two details led to an anti-phase correlation, as shown in Figure 11d. Furthermore, there were significant regions in the frequency range of 8–16 Hz during the 0–5 s period. As seen from Figure 10b, the significant regions of Set 2 were concentrated in the range of 2–16 Hz during the 0–10 s period due to the frequency variation of the transverse acceleration signal during train movement.

**Figure 12:**
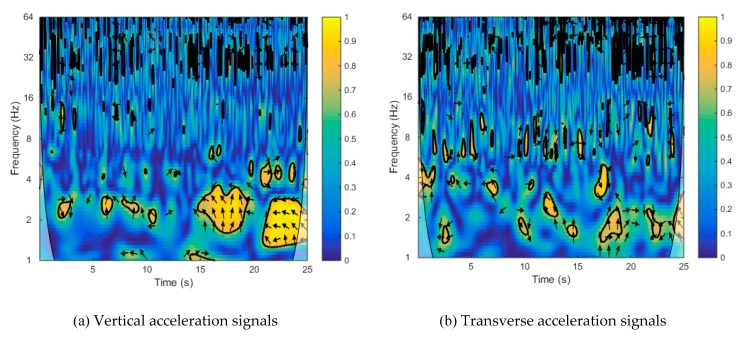
Wavelet coherence between the main span’s signals and the car body’s signals: (**a**) vertical acceleration signals (**b**) transverse acceleration signals.

The complex dynamic interactions of the train–bridge system led to difficulty regarding the assessment of each component’s influence on the dynamic responses of the whole system. In order to reveal the correlation between the dynamic performances of the train and the bridge, wavelet coherence was adopted to analyze the acceleration signals on the car body and at the center-point of the main span. Figure 12a illustrates that the significant regions in Set 3 mainly existed after 15 s, corresponding to the free vibration (i.e., when the train stopped on the bridge after braking) of both the train and the bridge, as shown in Figure 6a and Figure 8a. There were also some small, distributed, significant regions in the range of 2–4 Hz during the period where the train was traveling on the bridge (i.e., before 15 s). As shown in Figure 12b, the significant regions in Set 4 were scattered in the range of 1–8 Hz in the frequency domain. In addition, the dominant frequency of the free vibration part was about 2 Hz, which was in accordance with spectral analysis results of the main span’s transverse acceleration signal in the free vibration part. The wavelet coherence analysis results showed that the dynamic responses of the train and the bridge were not closely linked. This indicates that the stability of the vehicle is closely related to the dynamic performance of the vehicle itself rather than the structural dynamics of the bridge.

## 5. Conclusions

This paper investigated the dynamic responses of a metro train–bridge system under train-braking through field measurements. A continuous, three-span, rigid-frame bridge (42 m + 65 m + 42 m) and a standard B-type metro train were selected for experimentation on the elevated Metro Line 21 in Guangzhou (China). The acceleration signals at the center-points of the main span and one side-span were measured, as were as the acceleration signals of the car body and of the bogie frame. Due to the existence of different dominating frequencies in the train and bridge acceleration signals, Choi–Williams distribution (CWD) was used to minimize cross-term effects and maintain a high-quality time–frequency resolution. The results showed that Choi–Williams distribution provided more explicit results in the time–frequency domain. Wavelet coherence was used to investigate the relationship between the dynamic responses of the train and bridge in the time–frequency domain. The correlation of the dynamic responses between these two components was not closely linked during train-braking. This study provided a series of measured dynamic responses of the train–bridge system under train-braking, which could be used as a reference for further investigation regarding metro train–bridge systems.

## Figures and Tables

**Table 1:** Descriptions of the four sets of signals.

Set Number	Acceleration Signal 1	Acceleration Signal 2
Set 1	Vertical acceleration signals of the main span	Vertical acceleration signals of the side span
Set 2	Transverse acceleration signals of the main span	Transverse acceleration signals of the side span
Set 3	Vertical acceleration signals of the main span	Vertical acceleration signals of the car body
Set 4	Transverse acceleration signals of the main span	Transverse acceleration signals of the car body
